# *Acinetobacter calcoaceticus* is Well Adapted to Withstand Intestinal Stressors and Modulate the Gut Epithelium

**DOI:** 10.3389/fphys.2022.880024

**Published:** 2022-05-24

**Authors:** Janiece S. Glover, Brittney D. Browning, Taylor D. Ticer, Amy C. Engevik, Melinda A. Engevik

**Affiliations:** ^1^ Department of Regenerative Medicine and Cell Biology, Medical University of South Carolina, Charleston, SC, United States; ^2^ Department of Psychiatry and Behavioral Sciences, Medical University of South Carolina, Charleston, SC, United States; ^3^ Department of Microbiology and Immunology, Medical University of South Carolina, Charleston, SC, United States

**Keywords:** *Acinetobacter*, intestine, organoid, *Acinetobacter calcoaceticus*, metabolism

## Abstract

**Background:** The gastrointestinal tract has been speculated to serve as a reservoir for *Acinetobacter*, however little is known about the ecological fitness of *Acinetobacter* strains in the gut. Likewise, not much is known about the ability of *Acinetobacter* to consume dietary, or host derived nutrients or their capacity to modulate host gene expression. Given the increasing prevalence of *Acinetobacter* in the clinical setting, we sought to characterize how *A. calcoaceticus* responds to gut-related stressors and identify potential microbe-host interactions.

**Materials and Methods:** To accomplish these aims, we grew clinical isolates and commercially available strains of *A. calcoaceticus* in minimal media with different levels of pH, osmolarity, ethanol and hydrogen peroxide. Utilization of nutrients was examined using Biolog phenotypic microarrays. To examine the interactions of *A. calcoaceticus* with the host, inverted murine organoids where the apical membrane is exposed to bacteria, were incubated with live *A. calcoaceticus,* and gene expression was examined by qPCR.

**Results:** All strains grew modestly at pH 6, 5 and 4; indicating that these strains could tolerate passage through the gastrointestinal tract. All strains had robust growth in 0.1 and 0.5 M NaCl concentrations which mirror the small intestine, but differences were observed between strains in response to 1 M NaCl. Additionally, all strains tolerated up to 5% ethanol and 0.1% hydrogen peroxide. Biolog phenotypic microarrays revealed that *A. calcoaceticus* strains could use a range of nutrient sources, including monosaccharides, disaccharides, polymers, glycosides, acids, and amino acids. Interestingly, the commercially available *A. calcoaceticus* strains and one clinical isolate stimulated the pro-inflammatory cytokines *Tnf*, *Kc*, and *Mcp-1* while all strains suppressed *Muc13* and *Muc2*.

**Conclusion:** Collectively, these data demonstrate that *A. calcoaceticus* is well adapted to dealing with environmental stressors of the gastrointestinal system. This data also points to the potential for *Acinetobacter* to influence the gut epithelium.

## Introduction

*Acinetobacter* is a Gram-negative nonfermenting coccobacillus that is widely distributed in nature. Certain *Acinetobacter* species are classified as opportunistic pathogens and are commonly associated with healthcare associated infections (Peleg et al., 2008; Nemec et al., 2015; Mancilla-Rojano et al., 2020). The *Acinetobacter* species that are considered to be pathogens include *A. baumannii*, *A. pittii* and *A. nosocomialis* (Mancilla-Rojano et al., 2020). These species cause infections like bacteremia, ventilator-associated pneumonia, urinary tract infection, meningitis, and wound infection. *A. baumannii* is the most frequently isolated and best studied of the *Acinetobacter* species. In contrast to *A. baumannii*, *A. calcoaceticus* has been considered to have a lower virulence since colonization is more frequently noted than infection (Glew et al., 1977). However, *A. calcoaceticus* can still cause infection and understanding the mechanism by which *A. calcoaceticus* interacts with the host remains an important topic.

*Acinetobacter* species are capable of occupying several ecological niches, including the mammalian intestine. *Acinetobacter* species have been identified in the human fecal microbiota and it has been speculated that the gut could serve as a potential reservoir for *Acinetobacter* infection (Timsit et al., 1993; Xavier et al., 1996; Ayats et al., 1997; Dijkshoorn et al., 2005; Roy et al., 2010; Pandey et al., 2012; Aljindan et al., 2015; Cheng et al., 2015; Li et al., 2015; Braun et al., 2017; Li et al., 2019). Consistent with this hypothesis, *A. baumannii* can bind to rabbit small intestinal glycosphingolipids (Madar Johansson et al., 2020) and colonize the mouse gastrointestinal tract in a secretory IgA dependent manner (Coron et al., 2017; Ketter et al., 2018). Moreover, ampicillin treatment of mice has been shown to elevate *A. baumannii* and *A. calcoaceticus* fecal levels, suggesting that antibiotic selection could allow the outgrowth of endogenous strains (Raplee et al., 2021). Apart from these studies, little is known regarding the factors that influence *Acinetobacter* colonization and little data exists on the effects of *A. calcoaceticus*. The importance of *Acinetobacter* in the gut is highlighted by the fact that *Acinetobacter* species are elevated in certain disease states including ulcerative colitis, a subset of inflammatory bowel disease (IBD) (Gophna et al., 2006; Lucke et al., 2006; Leung et al., 2014; Kevans et al., 2015; Tang et al., 2015; Sjoberg et al., 2017; Sekido et al., 2020; He et al., 2021; Qi et al., 2022). In this study, we sought to examine the ability of *A. calcoaceticus* strains to survive environmental stressors found in the gut, interact with other gut microbes and utilize dietary components. We also examined the reciprocal interactions of *A. calcoaceticus* strains on the host using intestinal organoids.

## Methods

### General Bacterial Culturing Techniques

*Acinetobacter calcoaceticus* ATCC 23055 (American Type Culture Collection) and *A. calcoaceticus* CB1 (Carolina Biological Supply) were purchased from commercial sources. Four clinical isolates of *A. calcoaceticus* strains that were isolated from ventricular fluid of pediatric patients were provided by Dr. James Versalovic. All *A. calcoaceticus* strains were cultured aerobically in brain-heart-infusion (BHI) medium (ThermoFisher) at 37°C. Overnight cultures were sub-cultured into M9 minimal media containing glucose at an optical density (OD_600nm_) = 0.1. To model environmental stressors, M9 media was supplemented with NaCl (0.1, 0.5, or 1 M), H_2_O_2_ (0.05, 0.1, 0.2%), or ethanol (1, 2.5 or 5%). M9 was also adjusted to varying pH values (4, 5, 6, 7). Growth was monitored after 18 h incubation by OD_600nm._

To examine nutrient uptake, *A. calcoaceticus* strains were added to M9 media lacking glucose at OD_600nm_ = 0.1. Then 100 μL of this culture was added to each well of a 96-well Biolog Phenotype Microarrays for Microbial Cells (PM1 and PM2 plates). Growth was monitored at 18 h by OD_600nm_ on a Synergy HT BioTek plate reader. Stool-based bioreactors were generated as previously described (Engevik et al., 2021). Briefly, stool samples were cultured anaerobically for 24 h to allow microbes to establish stable communities, then inoculated with *A. calcoaceticus* strains at an OD_600nm_ = 0.05. After 48 h of incubation with *A. calcoaceticus* strains, samples were collected for gDNA isolation. For imaging purposes, *A. calcoaceticus* strains were fluorescently tagged with CFDA-SE (ThermoFisher) as previously described (Engevik et al., 2021). Briefly, *A. calcoaceticus* strains were washed 2x with sterile PBS and incubated for 1 h aerobically at 37°C with 10 μM CFDA-SE. After the incubation, bacteria were washed 3–5x with sterile PBS to remove any unused CFDA-SE. Bacteria were then ready for incubation with organoids.

### Organoid Generation

Intestinal organoids were generated from four adult (8–12 weeks) C57B6/J mice as previously described (Engevik et al., 2013). The jejunum was rapidly dissected, washed with ice-cold PBS (PBS; Gibco) and opened longitudinally. The jejunum was then cut into ∼1-cm length pieces and placed in a 15-ml conical tube containing 5 ml of ice-cold PBS, 43.4 mM sucrose, 0.5 mM DTT and 3 mM EDTA (Gibco). The tissue was incubated at 4°C rocking for 30 min. Crypts were mechanically disrupted by shaking in 5 ml of ice-cold PBS with D-sorbitol and sucrose and collected following filtration with a 70-µm cell strainer (Corning cat# 431,751). Crypts were centrifuged, resuspended in Matrigel (Corning), and incubated with complex media containing WNT, R-spondin, Noggin and EGF. Organoids were passaged >2 times to ensure no tissue fragments remained. Organoids were differentiated and grown inside-out by adding split organoids directly to complex media without Wnt and incubated at 37°C with 5% CO2 for 5 days before use.

For imaging purposes, live fluorescently tagged *A. calcoaceticus* was incubated with inside-out organoids for 3 h. Organoids were then washed with PBS, fixed with 4% paraformaldehyde for 30 min, incubated in 30% sucrose/PBS overnight and frozen embedded. Sections were stained with phospho-ezrin, radixin, moesin (1:200 dilution, Rabbit Ab, Cell Signaling #3726S) overnight at 4°C. After washing, sections were incubated with donkey-anti-rabbit Alexa Fluor 555 (1:1,000 dilution, ThermoFisher # A31572) for 1 h at room temperature and counter stained with Hoechst 33,342 (Invitrogen) for 10 min at room temperature. Slides were cover-slipped with mounting media (Life Technologies) and imaged using a Zeiss AxioScan at ×20 (Zeiss). The relative fluorescence intensity of adhered bacteria was quantified using FIJI (Formerly ImageJ) software (NIH) and reported as relative fluorescence. Three regions of interests per image and four images per slide were used for semi-quantitation of stain intensity.

For gene expression analysis, inside-out organoids were incubated with live *A. calcoaceticus* strains adjusted to an OD_600nm_ = 1.0 for 3 h at 37°C (*n* = 4 different mouse organoids; performed in replicates per mouse). After incubation, organoids were centrifuged at 300 × g for 5 min and the organoid pellet was resuspended in 400 μL TRIZOL. Organoids were stored in TRIZOL at −80°C until the RNA was isolated.

### RNA Isolation, cDNA Generation, gDNA Isolation and qPCR

Total RNA was extractr’ed from organoids in TRIZOL according to manufactures instructions, with the addition of glycogen. cDNA was generated from 500 ng RNA via the Verso cDNA synthesis kit (ThermoFisher #AB-1453). gDNA was isolated from 1 ml aliquots of stool-based bioreactors using the Zymo Quick-DNA Fecal/Soil Microbe Kits according to the manufacturer’s instructions. Quantitative real time PCR (qPCR) was performed using a Bio-Rad CFX96 Real Time qPCR machine (Bio-Rad). Forward and reverse primers were added to SYBR Green mastermix (Genesee Scientific #17-501DP) and cDNA. Epithelial genes were normalized to the housekeeping gene 18S and relative expression was calculated using the ddCT method. Bacterial colony forming units (CFUs) were calculated from CT values based on standard curves as previously described (Engevik et al., 2013).

### Statistics

Data are presented as mean ± standard deviation, with points representing individual bacteria strain growth rate with a n = 4 (repeated 3 independent times). Comparisons within groups were made with One-way Analysis of Variance (ANOVA) and comparisons between groups were made with a Two-way ANOVA (Table 1). All analyses were corrected for multiple comparisons by controlling the False Discovery Rate. GraphPad version 9.3 was used to generate graphs and statistics (GraphPad Software, Inc. La Jolla, CA). A *p < 0.05 value was considered significant while *n* is the number of experiments performed.

**TABLE 1:** Two Way ANOVA statistics from bacterial growth in the presence of stressors. All comparisons were made against *A. calcoaceticus* ATCC 23055. Significant *p* values (*p* < 0.05) are colored in blue.

	NaCl				pH					ETOH				H_2_0_2_		
Strain	0 M	0.1 M	0.5 M	1 M	pH 7	pH 6	pH 5	pH 4	0%	1%	2.50%	5%	0%	0.05%	0.10%	0.20%
CB1	0.1379	0.1097	<0.0001	<0.0001	0.0148	0.0003	0.4224	0.2573	0.079	<0.0001	<0.0001	0.0204	0.2819	0.163	0.2286	0.5514
M3	0.9269	0.4824	0.0126	0.0136	0.1814	0.3755	0.0133	0.0827	0.31	<0.0001	0.0023	0.0594	0.2781	0.0335	<0.0001	<0.0001
T82	0.2879	0.0218	0.9543	0.0013	0.034	0.7245	0.6418	0.4756	0.8335	0.0001	<0.0001	0.0003	0.2543	0.2975	0.0393	0.002
M5	0.577	0.0131	<0.0001	<0.0001	0.8767	0.0002	0.2652	0.2672	0.1845	<0.0001	0.0018	<0.0001	0.0775	0.0083	0.0734	0.0007
X75	0.1106	0.3661	<0.0001	0.0002	0.2573	0.0002	0.0003	0.0017	0.4996	<0.0001	<0.0001	<0.0001	0.0833	0.0199	<0.0001	<0.0001

## Results

**FIGURE 1:**
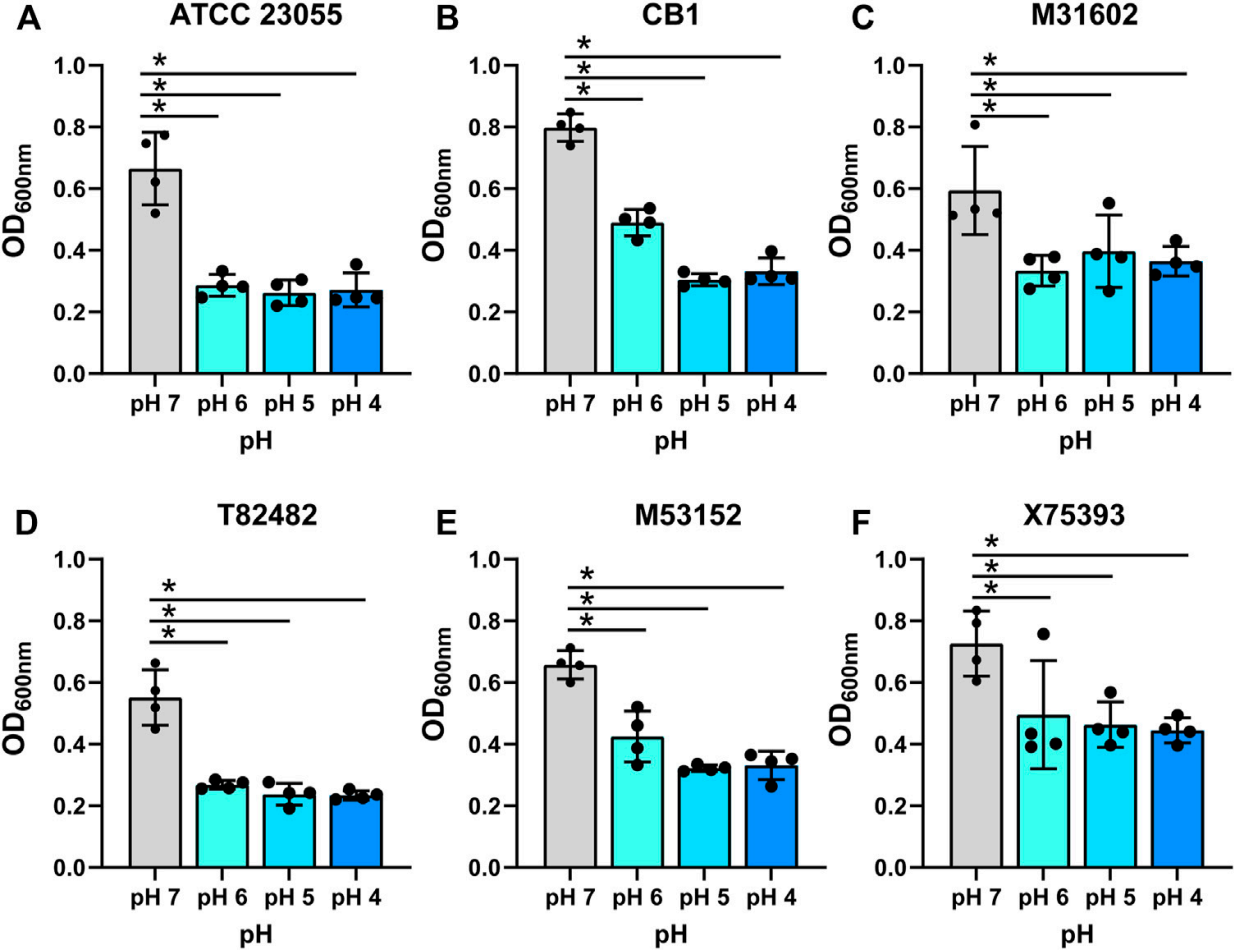
Growth of **(A)** *calcoaceticus* in various pHs. Growth of *A. calcoaceticus* strains as measured by OD_600nm_ after 18 h of incubation. Growth was examined in the following strains: **(A)** ATCC 23055, **(B)** CB1; clinical isolates: **(C)** M31602, **(D)** T82482, **(E)** M53152, and **(F)** X75,393). *p < 0.05, One Way ANOVA.

It has been speculated that the gut may be a site for *Acinetobacter* colonization and thus could be a source of endemic infections (Timsit et al., 1993; Xavier et al., 1996; Ayats et al., 1997; Dijkshoorn et al., 2005; Roy et al., 2010; Pandey et al., 2012; Aljindan et al., 2015; Cheng et al., 2015; Li et al., 2015; Braun et al., 2017; Li et al., 2019). To examine the efficiency of *A. calcoaceticus* strains to withstand the conditions of the gastrointestinal tract, we grew commercially available and clinical isolates of *A. calcoaceticus* in minimal media at pH 7, 6, 5, and 4. Cultures were seeded with OD_600n_ = 0.1 and growth was considered to be above an OD_600nm_ = 0.2. As expected, we observed robust growth of the commercially available *A. calcoaceticus* strains (ATCC 23055, CB1) and clinical isolates (M31602, T82482, M53152, and X75393) in pH 7 media (Figures 1A–F; Table 1). We observed growth (OD_600nm_ > 0.2) of all strains at pH 6, pH 5, and pH 4; although growth was significantly reduced compared to pH 7. These findings suggest that *A. calcoaceticus* is well adapted to withstand varying intestinal pHs.

**FIGURE 2:**
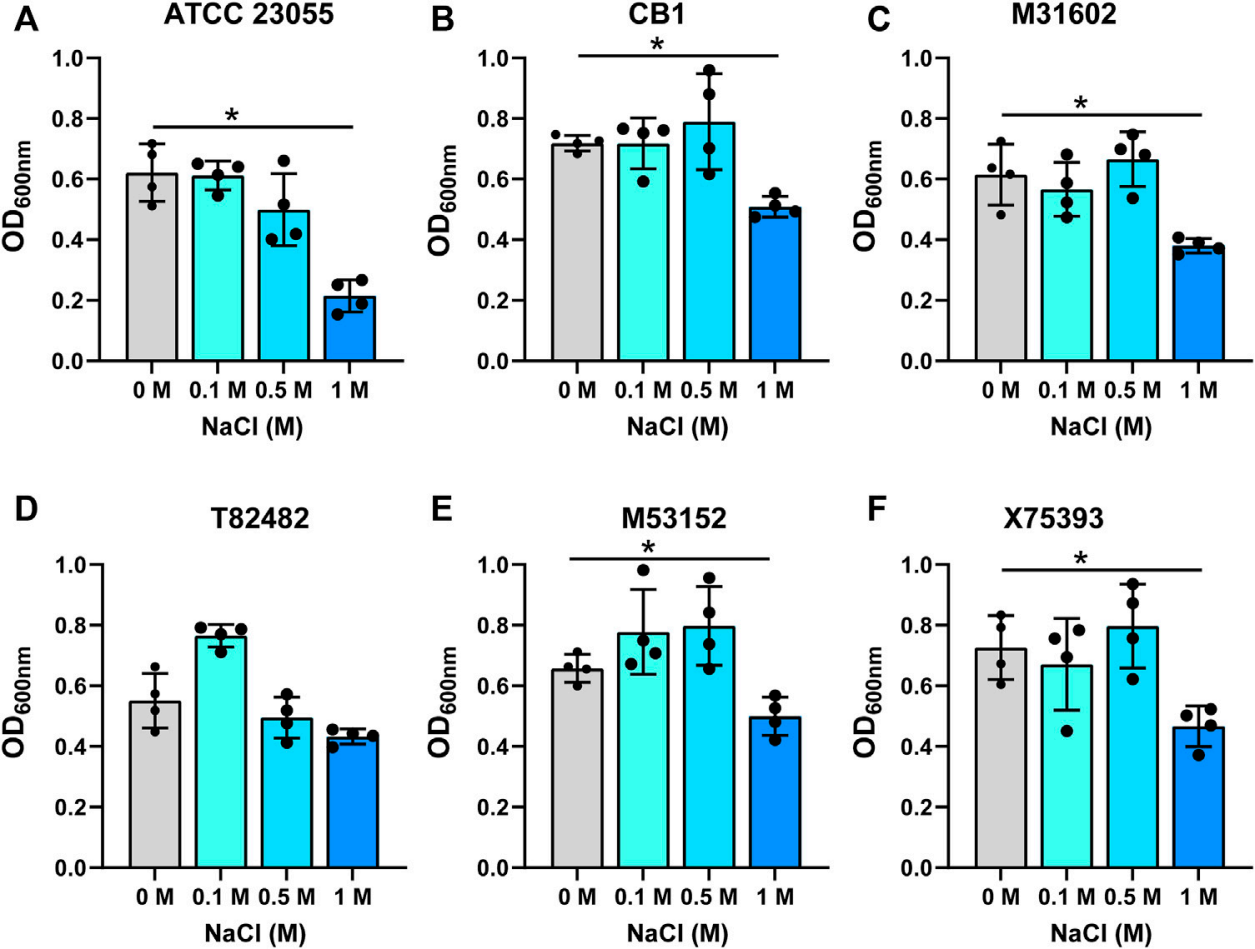
Growth of **(A)** *. calcoaceticus* at varying osmolarity. Growth of *A. calcoaceticus* strains as measured by OD_600nm_ after 18 h of incubation. Growth was examined in the following strains: **(A)** ATCC 23055, **(B)** CB1; clinical isolates: **(C)** M31602, **(D)** T82482, **(E)** M53152, and **(F)** X75,393). *p < 0.05, One Way ANOVA.

The intestine harbors varying ranges of osmolarity, with the small intestinal villi encountering 400–700 mM (Overduin et al., 2014). To model the gut, we added increasing concentrations of NaCl (0.1, 0.5 and 1 M) to minimal media and examined the growth of *A. calcoaceticus* (Figures 2A–F; Table 1). All strains were found to have similar growth at 0.1 and 0.5 M as media controls. Decreased growth was observed at 1 M NaCl with all but the T82482 strain. We observed that the ATCC 23055 strain exhibited the most significant decline in growth (Figure 2A). These data highlight that *A. calcoaceticus* can grow well in various osmolarities which mirror the gut environment.

**FIGURE 3:**
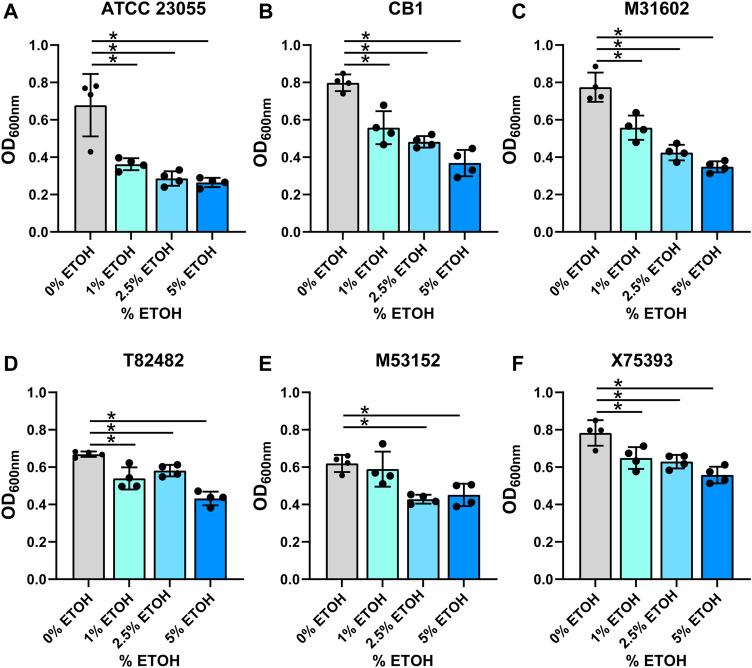
**(A)** *calcoaceticus* growth in percentages of ethanol. Growth of *A. calcoaceticus* strains as measured by OD_600nm_ after 18 h of incubation. Growth was examined in the following strains: **(A)** ATCC 23055, **(B)** CB1; clinical isolates: **(C)** M31602, **(D)** T82482, **(E)** M53152, and **(F)** X75,393). *p < 0.05, One Way ANOVA.

**FIGURE 4:**
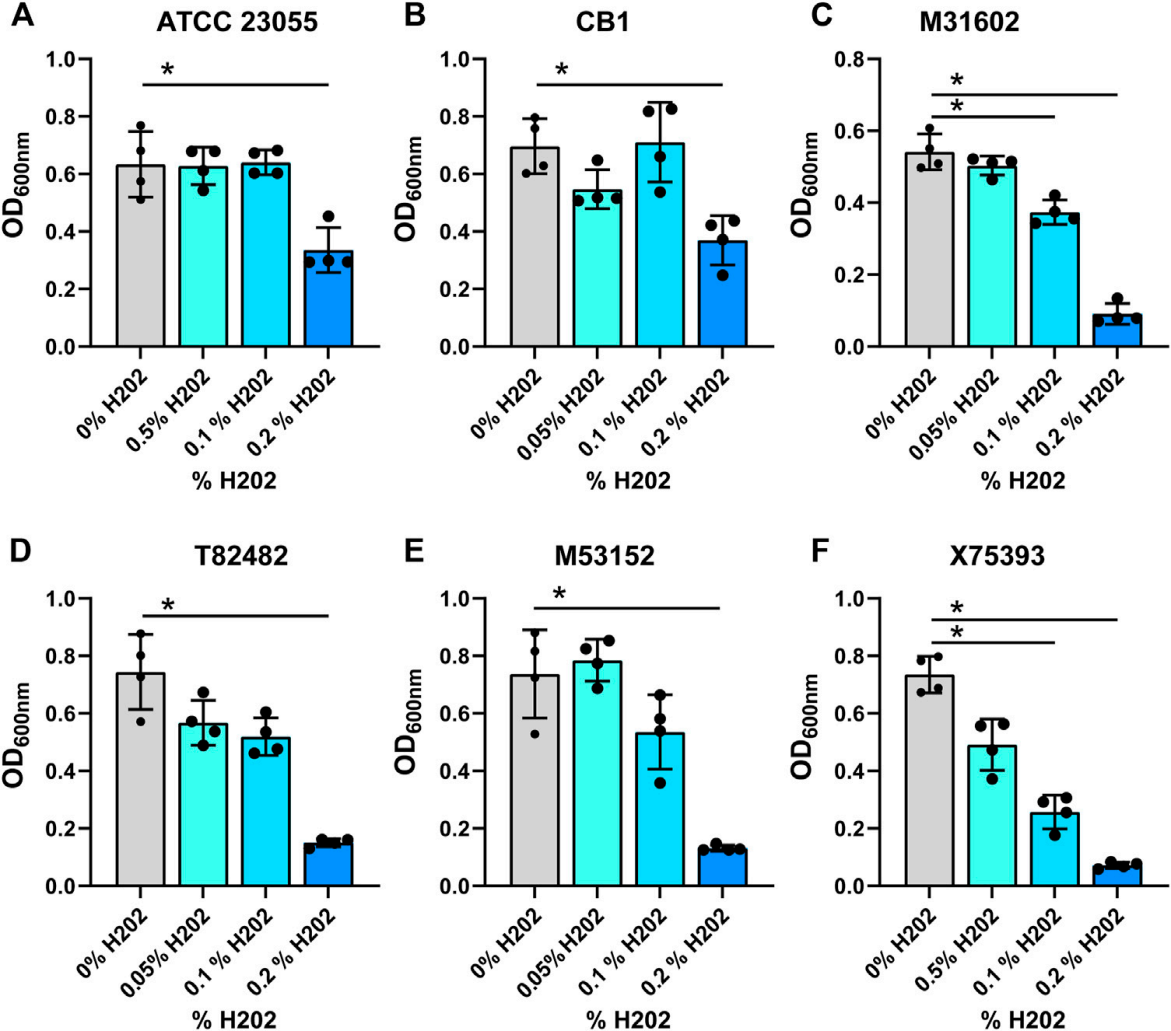
**(A)** *calcoaceticus* growth in hydrogen peroxide. Growth of *A. calcoaceticus* strains as measured by OD_600nm_ after 18 h of incubation. Growth was examined in the following strains: **(A)** ATCC 23055, **(B)** CB1; clinical isolates: **(C)** M31602, **(D)** T82482, **(E)** M53152, and **(F)** X75,393). *p < 0.05, One Way ANOVA.

In the gut, commensal microbes such as Lactobacilli can generate ethanol and hydrogen peroxide (Engevik and Versalovic, 2017) and microbes occupying the same niche must adapt to these stressors. To model the production of localized ethanol and hydrogen peroxide, we supplemented minimal media with ethanol (1, 2.5 or 5%) (Figures 3A–F; Table 1) or hydrogen peroxide (0.05, 0.1 or 0.2%) (Figures 4A–F; Table 1). Impressively, all *A. calcoaceticus* strains could grow in up to 5% ethanol. Our commercially available ATCC 23055 strain was the most sensitive strain to ethanol, exhibiting a ∼2-fold decrease in growth in 1% ethanol compared to media controls (Figure 3A). In contrast, clinical isolates T82482 (Figure 3D), M53152 (Figure 3E), and X75,393 (Figure 3F) exhibited only a slight decline in growth at the higher ethanol concentrations; suggesting that these clinical isolates are highly resistant to ethanol. When *A. calcoaceticus* was treated with hydrogen peroxide (Figures 4A–F), all strains could grow with 0.05 and 0.1% hydrogen peroxide. The commercially available ATCC 23055 and CB1 were the most tolerant to 0.2% hydrogen peroxide (Figures 4A,B); exhibiting a less than 2-fold decrease in growth compared to the no hydrogen peroxide controls. In contrast, the clinical isolates M31602 (Figure 4C), T82482 (Figure 4D), M53152 (Figure 4E), and X75,393 (Figure 4F) were sensitive to 0.2% hydrogen peroxide and did not grow above the seeded density of OD_600nm_ = 0.1. Collectively, these data with stressors indicate that *A. calcoaceticus* is well adapted for the environmental stressors associated with gut colonization.

**FIGURE 5:**
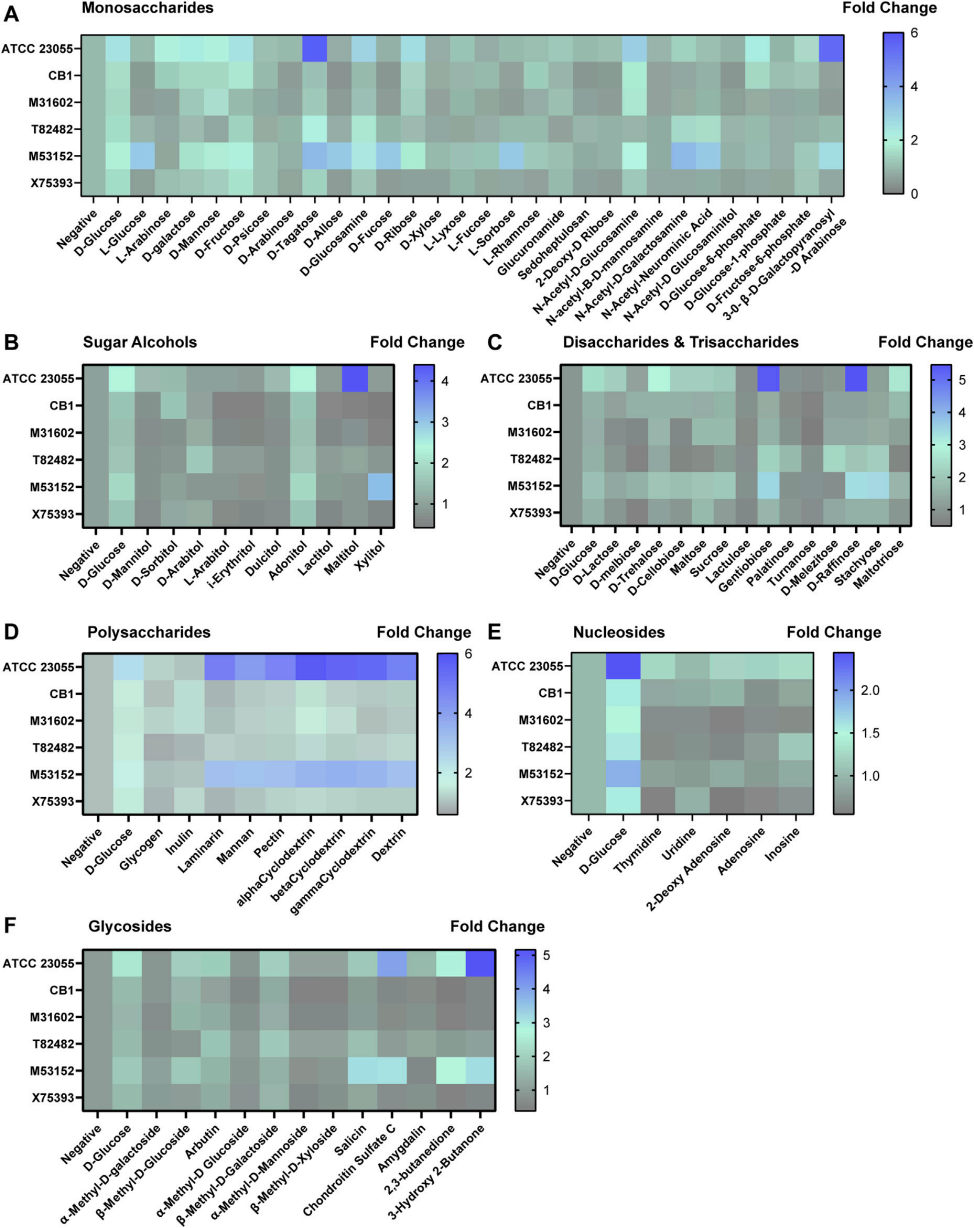
*Acinetobacter* growth in varying carbon sources. Heat maps representing fold change in growth with the negative control (no added nutrients) set at 1. Growth was examined with **(A)** monosaccharides, **(B)** sugar alcohols, **(C)** disaccharides and trisaccharides, **(D)** polysaccharides, **(E)** nucleosides and **(F)** glycosides after 18 h incubation.

**FIGURE 6:**
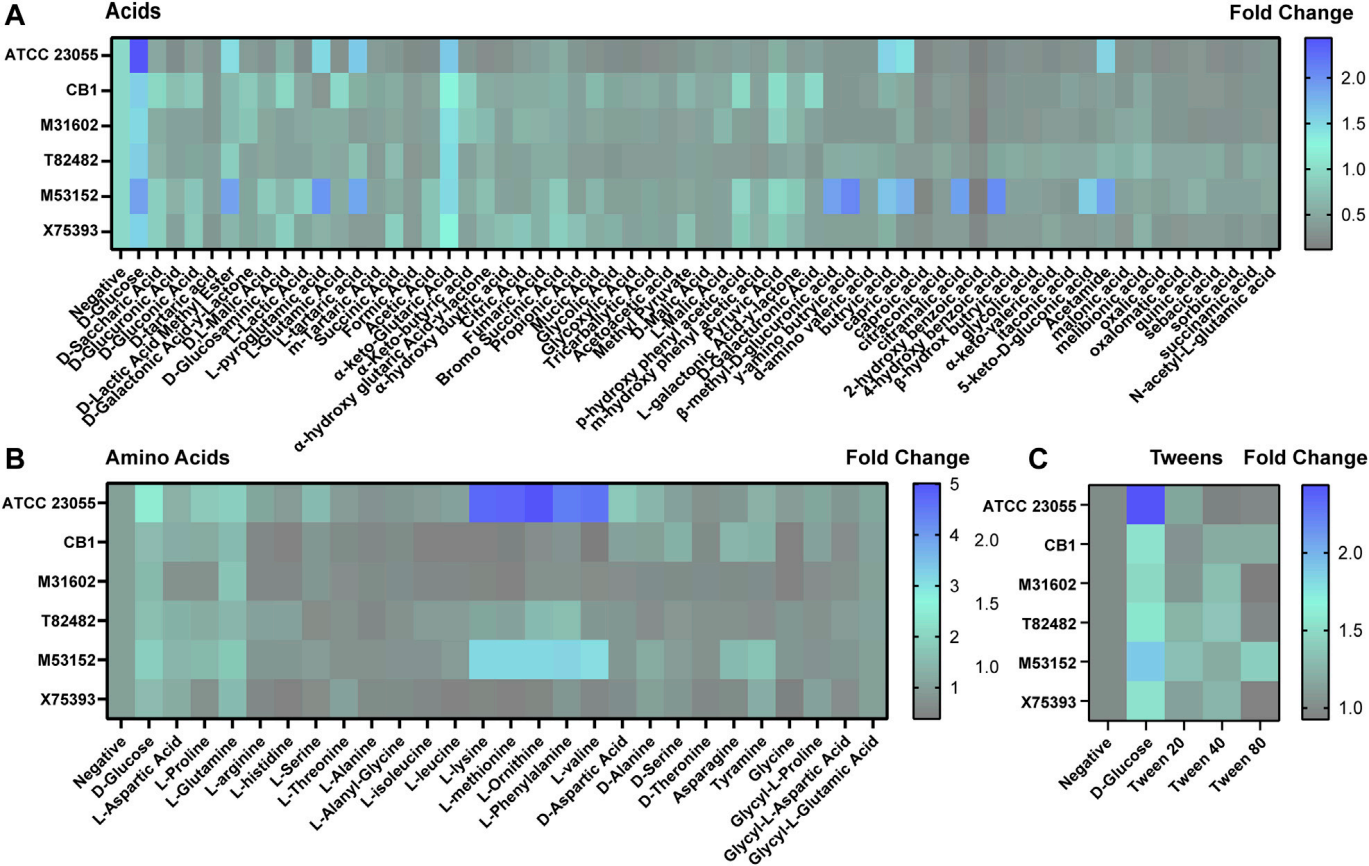
*Acinetobacter* growth in acids, amino acids and tweens. Heat maps representing fold change in growth with the negative control (no added nutrients) set at 1. Growth was examined with **(A)** acids, **(B)** amino acids, and **(C)** tweens after 18 h incubation.

Next, we sought to examine the potential nutrient sources for *A. calcoaceticus* within the intestine. To address this, we grew *A. calcoaceticus* strains in minimal media lacking glucose in Biolog phenotypic microarrays (Figures 5, 6). Growth was considered to be > 1.5 fold change. Compared to growth in a media without a carbon source, *A. calcoaceticus* had improved growth in the presence of glucose (Figure 5A). Since *A. calcoaceticus* had improved growth with glucose, we first examined monosaccharides (Figure 5A). ATCC 23055 and CB1 grew well with L-arabinose, D-galactose, D-mannose, D-fructose, D-tagatose, D-glucosamine, D-ribose, and N-acetyl-D-glucosamine. Interestingly, clinical isolates M31602, T82482 and M53152 did not grow with arabinose and exhibited strain-dependent growth with D-mannose, D-fructose, S-tagatose, D-ribose and N-acetyl-D-glucosamine. M53152 alone grew with L-glucose, B-D-allose, D-fucose, and L-sorbose, highlighting strain-specific nutrient preferences. When we examined alcohol sugars (Figure 5B), we found that all strains could use adonitol and no strains could use D-mannitol, L-arabitol, i-erythritol, or dulcitol.

In terms of growth with disaccharides (Figure 5C), we found that all strains used gentiobiose and no strains used lactulose or turnanose. For the other disaccharides and trisaccharides, we observed strain-dependent growth. For example, M53152 grew with D-melbiose, D-trehalose, maltose, D-melibiose, sucrose, D-cellbiose, D-raffinose, stachyose and maltotriose, while T82482 only grew with D-palatinose, D-melezitose, D-raffinose and stachyose. When we examined polysaccharide utilization, we found very similar profiles between ATCC 23055 and M53152. Both strains were highly efficient at using laminarin, mannan, pectin, α-Cyclodextrin, β-Cyclodextrin, γ-cyclodextrin and dextrin (Figure 5D). In general, most strains grew well with dietary polysaccharides. In the nucleosides (Figure 5E) and glycosides (Figure 5F) classification, we found strain-dependent use of specific compounds. ATCC 23055 and M53152 exhibited the highest fold change in growth with chondroitin sulfate C, 2,3-butanedione and 3-hydroxy 2-butanone.

Of the acids (Figure 6A), we found that all strains could use a-keto-glutaric acid. ATCC 23055 and M53152 responded to the largest number of acids, including B-methyl-D-glucuronic acid, γ-amino butryic acid, butryic acid, capric acid, 4-hydroxy benzoic acid and acetamide. Unique profiles were observed with the other acids examined depending on the strain. Even without a carbon source, we found that all *A. calcoaceticus* strains had improved growth with L-glutamine and ATCC 23055 and M53152 used L-lysine, L-methionine, L-Ornithine, L-Phenylalanine and L-valine (Figure 6B), indicating that select amino acids could be used as an alternative to carbon. Tween can be employed as a stool emulsifier and tween enemas have been used to treat fecal mass obstructions (Wood and Katzberg, 1978). None of the *A. calcoaceticus* strains responded to tween with improved growth (Figure 6C). These data indicate that *A. calcoaceticus* can use a wide range of nutrients sources, ranging from sugars to amino acids.

**FIGURE 7:**
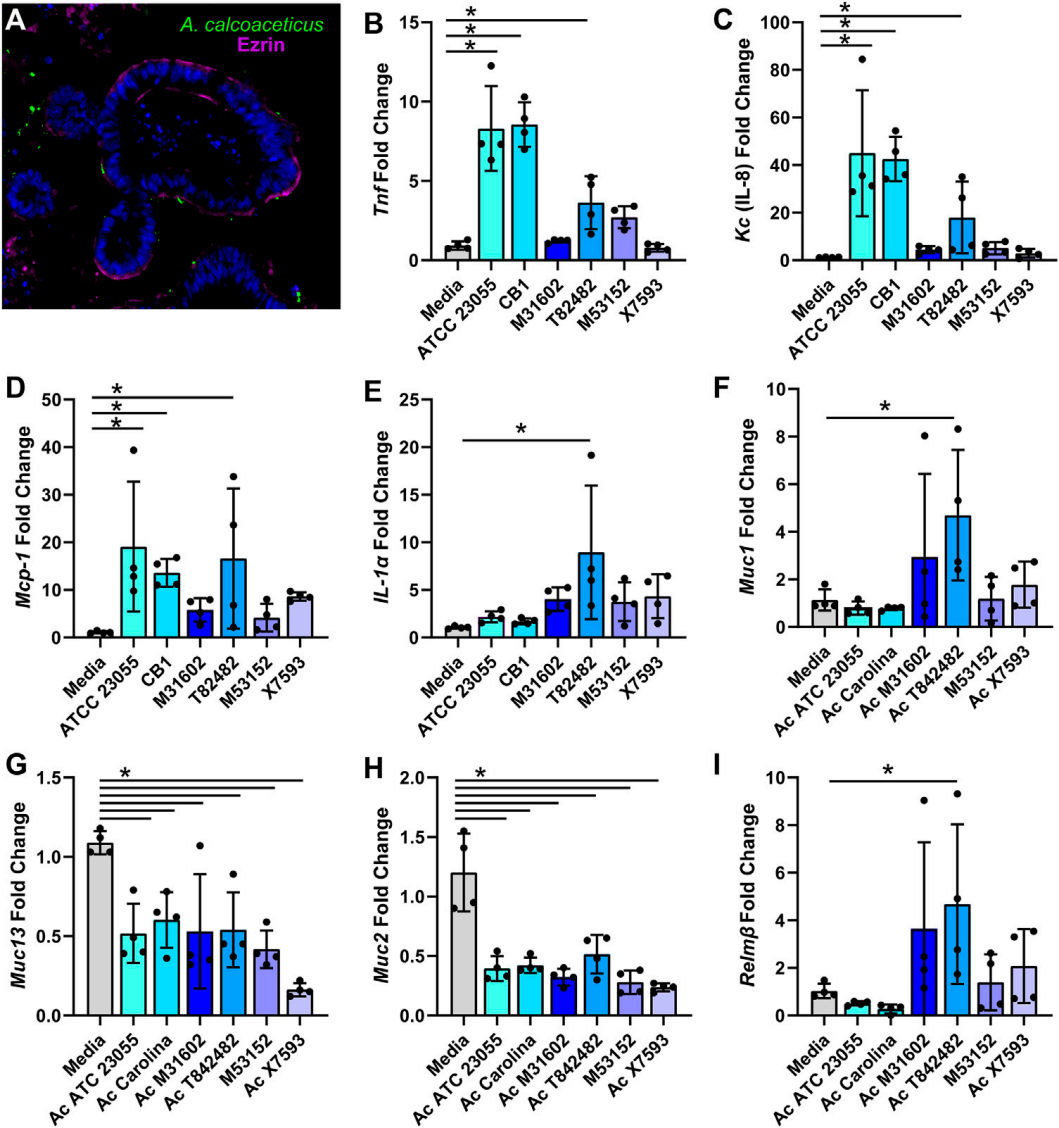
**(A)** *calcoaceticus* induces inflammatory responses in intestinal organoids. **(A)** Inside-out organoids incubated with live fluorescently tagged *A. calcoaceticus* and immunostained with the apical marker phospho-Ezrin. qPCR analysis of organoids after 3 h of incubation examining expression of **(B)** *Tnf*, **(C)** *IL-18*, **(D)** *Mcp-1*, **(E)** *IL-1alpha*, and **(F)** *Muc1*, **(G)** *Muc13*, **(H)** *Muc2*, and **(I)** *Relmβ.*

In addition to stressors and nutrients, *Acinetobacter* species encounter a complex community of micro-organisms when colonizing the gastrointestinal tract. To confirm that *A. calcoaceticus* could proliferate in this competitive environment, we cultured stool-based bioreactors and introduced *A. calcoaceticus* strains. After 48 h of culturing, we examined the presence of *A. calcoaceticus* in the bioreactors by qPCR (Supplementary Figure S1A). We found that all strains effectively colonized the bioreactors, indicating that *A. calcoaceticus* could be present within the intestinal milieu. Next, we sought to examine how *A. calcoaceticus* interacted with the intestinal epithelium. To test whether *A. calcoaceticus* was able to stimulate epithelial responses, we incubated live *A. calcoaceticus* strains with apical side-out intestinal organoids for 3 h. By immunostaining, we found that some *A. calcoaceticus* microbes adhered to the organoids (Figure 7A). A similar level of bacteria was observed on all organoids regardless of the strain (Supplementary Figure S1B). Analysis of pro-inflammatory cytokines by qPCR revealed that the commercially available strains ATCC 23055 and CB1 and one of our clinical isolates T82482 increased the expression of *Tnf* (Figure 7B), *Kc* (Figure 7C) and *Mcp-1* (Figure 7D) compared to media controls. The T82482 strain also increased the expression of *IL-1α* (Figure 7E). Inflammation is known to regulate intestinal mucus, so we also examined adherent mucins *Muc1* and *Muc13* and secreted mucins *Muc2* in our organoid model. T82482 was the only strain that upregulated *Muc1* (Figure 7F), but all strains suppressed *Muc13* levels (Figure 7G) compared to media controls. Likewise, all strains suppressed *Muc2* expression (Figure 7H). We also examined an antimicrobial protein secreted by goblet cells, Relmβ, and we observed that *Relmβ* expression was increased in response to T82482 (Figure 7I), suggesting that goblet cells were not decreased in this model despite decreased *Muc2*. Together these findings highlight that *A. calcoaceticus* strains are adept at dealing with environmental stressors, are able to consume multiple nutrient sources, colonize in the presence of other microbes, and can elicit pro-inflammatory signaling pathways.

## Discussion

The digestive tract is proposed to be a reservoir for *Acinetobacter* spp. (Timsit et al., 1993; Corbella et al., 1996; Agusti et al., 2002; Dijkshoorn et al., 2005; Thom et al., 2010; Lim et al., 2014). In this study, we confirmed the ability of *A. calcoaceticus* strains to withstand conditions that recapitulate the gastrointestinal tract luminal environment and use various nutritional sources found in the intestine. We identified that *A. calcoaceticus* strains are fairly resistant to changing pH, osmolarity, ethanol and hydrogen peroxide levels. We also found that the majority of *A. calcoaceticus* strains used glucose, L-arabinose, D-galactose, D-mannose, D-fructose and N-acetyl-D-glucosamine, D-trehalose, adonitol, mannan, pectin, α-Cyclodextrin, β-Cyclodextrin, γ-cyclodextrin, dextrin, D-ribose and α-keto-glutaric acid and L-glutamine. These data indicate that *A. calcoaceticus* can use a wide range of nutrients sources. Bioreactor experiments confirmed that *A. calcoaceticus* could colonize with other gut microbes. This work adds to existing research and suggests that *Acinetobacter* spp. are well adapted for survival in the gastrointestinal tract.

Entry of *Acinetobacter* spp. into the gut has recently been examined by Coron et al. (2017). The authors found that intranasal administration of *A. baumannii* in mice, which mimicked the major method by which ventilated patients in ICUs commonly become infected with *Acinetobacter*, resulted in digestive-tract colonization. This data suggests that patients could become colonized with *Acinetobacter* spp. in hospital settings and this gut colonization could be the precursor of severe infections. Consistent with this notion, Corbella *et al.* identified that patients colonized with *A. baumannii* in their digestive system had a positive association for blood infections with multidrug-resistant *A. baumannii* strains compared to patients without colonization (Corbella et al., 1996). Similarly, Medina *et al.* found that *A. baumannii* gut colonization was an independent risk factor for the development of *A. baumannii* respiratory infections (Medina-Presentado et al., 2013). Wisplinghof *et al.* reported that the portal of entry was not identified in 48.6% of the *A. baumannii* bloodstream infections, suggesting that a significant proportion of these infections could be due to intestinal carriage (Wisplinghoff et al., 2000; Coron et al., 2017). Our work complements these findings by demonstrating that *Acinetobacter* species are equipped to colonize the gastrointestinal tract, where they could serve as a reservoir for infection.

In animal models, *A. baumannii* colonized both the small and large intestine (Coron et al., 2017; Ketter et al., 2018). In these models, intestinal inflammation was not specifically examined. However, our apical inside-out organoid model revealed that certain strains of *A. calcoaceticus* stimulated pro-inflammatory cytokines. This data can be interpreted in several ways. First, it is possible that in the setting of a complex gut microbiota *in vivo*, other microbes may dampen pro-inflammatory signatures associated with *Acinetobacter*. Second, the existing *in vivo* studies used *A. baumannii* and this study focused on *A. calcoaceticus*. It is therefore possible that differences may exist between the species. Third, our data suggests that pro-inflammatory responses are strain dependent and these strain differences may also exist for *A. baumannii* strains. Future studies using mouse models are warranted to fully dissect the colonization capacity and epithelial crosstalk with *A. calcoaceticus*.

Elevated levels of *Acinetobacter* in the setting of IBD has been observed in several studies (Gophna et al., 2006; Lucke et al., 2006; Leung et al., 2014; Kevans et al., 2015; Tang et al., 2015; Sjoberg et al., 2017; Sekido et al., 2020; He et al., 2021; Qi et al., 2022). Two studies found high abundance of *Acinetobacter* in pediatric patients with newly diagnosed ulcerative colitis (Kevans et al., 2015; Sjoberg et al., 2017); providing potential evidence that *Acinetobacter* species could be contributing to the onset of intestinal inflammation in genetically susceptible patients. Another study found that *Acinetobacter* was enriched in the mucosa-associated bacteria during active colitis in ulcerative colitis patients (Tang et al., 2015). This study found that *Acinetobacter* levels significantly correlated with microbial pathways in actively inflamed colitis tissue, suggesting a potential causal relationship between *Acinetobacter* and intestinal inflammation. Another study identified *Acinetobacter* in the CD14^+^CD11c + CD163low subset macrophages in the lamina propia of ulcerative colitis patients (Sekido et al., 2020), which indicates these microbes were able to bypass the epithelial barrier. In our intestinal organoid model, we found that several *A. calcoaceticus* strains stimulated pro-inflammatory cytokine expression and suppressed mucin production. These findings mirror what has been observed in ulcerative colitis patients (Trabucchi et al., 1986; Raouf et al., 1992; Pullan et al., 1994; Tytgat et al., 1996; Hanski et al., 1999; Heazlewood et al., 2008; Zhao et al., 2010; Larsson et al., 2011; Antoni et al., 2014; Johansson et al., 2014; Wenzel et al., 2014). Our findings provide further evidence for the potential link between *Acinetobacter*, intestinal inflammation and IBD. In the future, we plan to dissect the mechanisms of how *A. calcoaceticus* initiates inflammation in more depth.

Another observation from our organoid model was that not all the clinical isolates stimulated pro-inflammation cytokine expression. Gram-negative bacteria such as *Acinetobacter* can activate TLR4 on host cells via the cell wall component lipopolysaccharide (LPS) (Pelletier et al., 2013). LPS is comprised of lipid A, the core oligosaccharide, and the O-specific antigen. Lipid A is considered the bioactive component of LPS and is responsible for activating immune responses (Pelletier et al., 2013). *A. baumannii* has been shown to modify their lipid A with the addition of positively charged residues including ethanolamine, phosphoethanolamine, aminoarabinose, and glucosamine (Moskowitz et al., 2004; Arroyo et al., 2011; Basheer et al., 2011; Beceiro et al., 2011; Llobet et al., 2011; Pelletier et al., 2013). These modifications enhance the resistance of *A. baumannii* to the antibiotic colistin and suppresses their immunostimulatory capacity (Pelletier et al., 2013). In addition to modifying LPS, some clinical strains of *A. baumannii* have been identified with loss-of-function mutations in genes in the LPS biosynthetic pathway (Moffatt et al., 2010; Nagy et al., 2019); resulting in strains which completely lack LPS. Although *A. baumannii* can survive in the absence of LPS, these microbes have distinct morphological defects and growth alterations under laboratory conditions (Nagy et al., 2019; Beceiro et al., 2011; Bojkovic et al., 2015; Powers et al., 2018; Boll et al., 2016). All of our *A. calcoaceticus* strains were resistant to colistin (*data not shown*) suggesting that some LPS modification might have occurred in these microbes. Since we didn’t observe significant morphological or growth differences between our strains, we think that all our stains harbor LPS, but we speculate that their may be different modifications between our *A. calcoaceticus* strains which could account for the variability in cytokine stimulation. We plan in future studies to examine the LPS structures of our *A. calcoaceticus* strains.

Interestingly we noted differences between our commercially available strains and clinical isolates in many of our results. For example, the clinical isolates were more adapted at survival in high concentrations of ethanol and NaCl than the lab adapted strains. The growth of the clinical isolates was also not as robust as the ATCC 23055 strain in utilizing many of the carbon sources, such as glucose, L-arbinose, D-trehalose, GluNAc, galactose, mannose and fructose. In our organoid model, we found that lab adapted ATCC 23055 and CB1 and one clinical isolate T82482 stimulate multiple pro-inflammatory cytokines, while the other strains had minimal stimulation of cytokines. A number of groups have begun to question the adequacy of laboratory-adapted reference strains to represent “real world” pathogenesis (Fux et al., 2005). Some laboratory strains have been sub-cultured for years, which may result in the loss of important pathophysiological characteristics or the dependence on lab-specific media components. This limitation can be overcome by including multiple strains, including clinical isolates, and examining their collective behavior. While we did note several differences, in general we found that all strains were fairly resistant to environmental stressors (pH, osmolarity, ethanol, and hydrogen peroxide) and we found several common nutrient sources across strains. Based on these studies, we believe that *A. calcoaceticus* species are well adapted to colonize the gastrointestinal tract and can consume a variety of nutritional sources. We also believe this work highlights the benefits of incorporating clinical isolates into future work.

There are several strengths in our study. To the best of our knowledge, this is the only study that has examined the ability of *A. calcoaceticus* to withstand conditions of the gastrointestinal tract and the first show that *A. calcoaceticus* can colonize human stool communities and modulate the gut epithelium. We incorporated several clinical isolates, which has allowed us to identify some global attributes of *A. calcoaceticus*. However, there are also several limitations. This work was all done *in vitro* and *in vivo* studies are necessary to truly identify the colonization capacity of *A. calcoaceticus*. Although we hypothesize that *A. calcoaceticus* activates TLR4 on the gut epithelium to drive inflammatory signatures, this work did not identify a specific mechanism and more work is needed to delineate how *A. calcoaceticus* modulates the intestinal epithelium and immune cells.

In summary, we demonstrate that *A. calcoaceticus* strains can withstand intestinal conditions and thrive with several dietary sources. We believe these attributes make *Acinetobacter* spp. ecologically fit for colonizing the gut. This information is clinically important since the gut likely serves as a reservoir for secondary *Acinetobacter* spp. infections. Thus, it might be possible to prevent secondary infections, like blood stream infections or pneumonia, by inhibiting *Acinetobacter* gut colonization and we believe this is an exciting area for future studies.

## Data Availability

The raw data supporting the conclusion of this article will be made available by the authors, without undue reservation.
