# Supplementary figures and images for: Acinetobacter calcoaceticus is Well Adapted to Withstand Intestinal Stressors and Modulate the Gut Epithelium
Source: Front Physiol. 2022 May 24;13:880024. doi: 10.3389/fphys.2022.880024 (PMC9170955; doi:10.3389/fphys.2022.880024)

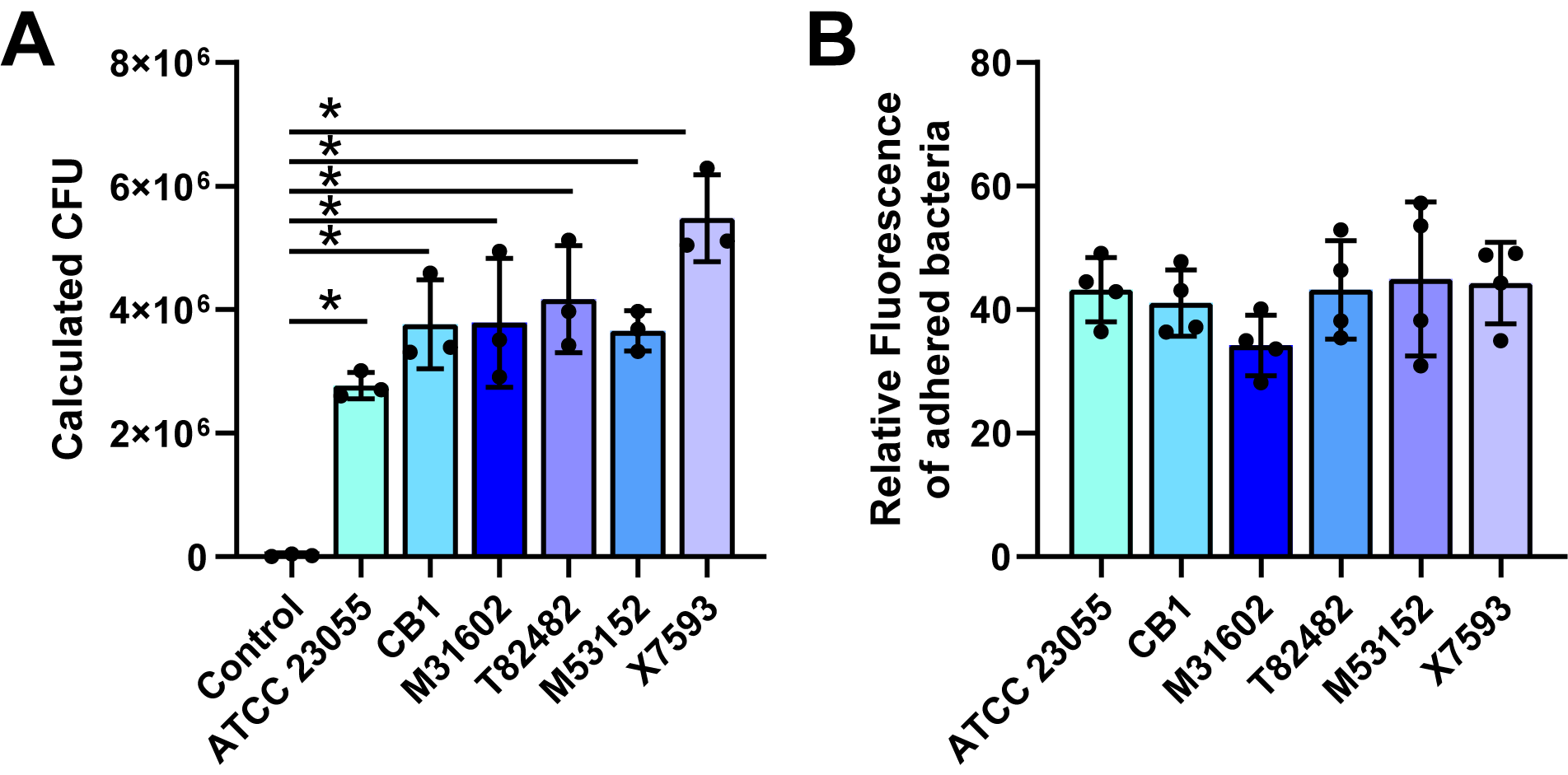

Supplement: Supplementary file 1 [file Image1.tif]
